# Risk Profiles for Weight Gain among Postmenopausal Women: A Classification and Regression Tree Analysis Approach

**DOI:** 10.1371/journal.pone.0121430

**Published:** 2015-03-30

**Authors:** Su Yon Jung, Mara Z. Vitolins, Jenifer Fenton, Alexis C. Frazier-Wood, Stephen D. Hursting, Shine Chang

**Affiliations:** 1 Translational Sciences Section, Jonsson Comprehensive Cancer Center, School of Nursing, University of California Los Angeles, Los Angeles, CA, United States of America; 2 Department of Epidemiology and Prevention, Division of Public Health Sciences, Wake Forest School of Medicine, Winston-Salem, North Carolina, United States of America; 3 Department of Food Science and Human Nutrition, Michigan State University, East Lansing, Michigan, United States of America; 4 USDA/ARS Children’s Nutrition Research Center, Department of Pediatrics, Baylor College of Medicine, Houston, Texas, United States of America; 5 Department of Nutritional Sciences, The University of Texas at Austin, Austin, Texas, United States of America; Harvard Medical School, UNITED STATES

## Abstract

**Purpose:**

Risk factors for obesity and weight gain are typically evaluated individually while “adjusting for” the influence of other confounding factors, and few studies, if any, have created risk profiles by clustering risk factors. We identified subgroups of postmenopausal women homogeneous in their clustered modifiable and non-modifiable risk factors for gaining ≥ 3% weight.

**Methods:**

This study included 612 postmenopausal women 50–79 years old, enrolled in an ancillary study of the Women's Health Initiative Observational Study between February 1995 and July 1998. Classification and regression tree and stepwise regression models were built and compared.

**Results:**

Of 27 selected variables, the factors significantly related to ≥ 3% weight gain were weight change in the past 2 years, age at menopause, dietary fiber, fat, alcohol intake, and smoking. In women younger than 65 years, less than 4 kg weight change in the past 2 years sufficiently reduced risk of ≥ 3% weight gain. Different combinations of risk factors related to weight gain were reported for subgroups of women: women 65 years or older (essential factor: < 9.8 g/day dietary factor), African Americans (essential factor: currently smoking), and white women (essential factor: ≥ 5 kg weight change for the past 2 years).

**Conclusions:**

Our findings suggest specific characteristics for particular subgroups of postmenopausal women that may be useful for identifying those at risk for weight gain. The study results may be useful for targeting efforts to promote strategies to reduce the risk of obesity and weight gain in subgroups of postmenopausal women and maximize the effect of weight control by decreasing obesity-relevant adverse health outcomes.

## Introduction

An increasing prevalence of obesity has emerged as a major public health concern, especially in older adults.[1,2] Particularly, in the United States, postmenopausal women older than 50 years, compared to premenopausal women, have a higher prevalence rate of obesity, as 47.6% of postmenopausal women are overweight, among whom over 25% are obese.[1,2] Obesity is a known risk factor for developing metabolic disorders such as metabolic syndrome, diabetes mellitus, cardiovascular diseases and hypothyroidism, stroke, inflammatory diseases, and gynecologic cancer.[1,3–6] In postmenopausal women, obesity is also associated with a higher risk of death from all causes and death from specific causes including coronary heart disease and breast cancer.[7–10] Additionally, in older adults, a higher fat mass is a predictor of a lower physical functioning and a higher frequency of disability and dependency.[11] Previous studies reported that in older people, a moderate weight loss (5%-10% of the initial body weight) improves obesity-related cardiovascular and metabolic abnormalities and all-cause mortality[12]; however, weight gain after menopause is associated with weight regain within a few years after weight-loss program.[3] Moreover, a recent study indicated that in older adults, weight gain rather than static measure of weight status (i.e., baseline body mass index [BMI]) is more predictive of mortality[7], which suggests the importance of identifying attributable factors to prevent excessive adiposity.

The contributing factors to increased postmenopausal adiposity encompass genetic, physiologic, psychosocial, and behavioral characteristics. The growing evidence from previous studies has indicated that in addition to genetic predisposition itself, environmental characteristics may interact with a genetic susceptibility, resulting in obesity.[2,13] In postmenopausal women, obesity-related modifiable and non-modifiable characteristics include physiologic factors such as aging, which is related to lower basal metabolic rate, sex-hormonal changes[1,2,14,15], and co-morbidities[5]; psychosocial factors including sleep disturbance[5], depression, and stress[16,17]; behavioral factors such as less physical activity and more sedentary lifestyles contributing to low energy expenditure[2,4,14,15,18], increased caloric intake[1], lower intake of dietary fiber[4,19–22] and low-fat diet[23,24], alcohol intake[4,13,25], and smoking.[2,4,26]

Although multiple studies evaluating risk factors individually to predict postmenopausal obesity are helpful, more comprehensive approaches are needed to examine the extent to which these factors co-act and interact to develop postmenopausal obesity and weight gain risk profiles. Research which accounts for these multiple interactions, while ascertaining the most predictive risk factors for obesity and weight gain, will give rise to more accurate clinical risk prediction model. Moreover, to our knowledge, no studies have explored interactions among obesity-relevant factors in subsets of postmenopausal women (e.g., younger vs. older women; black vs white women). Therefore, identifying the most relevant risk factors could prove to be useful to target efforts to reduce risk of obesity and weight gain in diverse subgroups of postmenopausal women.

Classification and regression tree (CART) analysis is an innovative approach which uncovers complex interactions among variables that may be overlooked in traditional analyses. [11,27] The CART methodology allows us to identify homogeneous subgroups of individuals based on shared factors related to an outcome of interest (i.e., ≥3% weight gain in our study) and examine the risk magnitude of given risk factors within the subgroup.[27,28] We employed this analytic approach to generate profiles of postmenopausal women who are at risk of 3% or more of weight gain after 3 years of follow-up, based on a set of modifiable and non-modifiable characteristics that have been known to be independently associated with postmenopausal obesity.

## Materials and Methods

### Study population

The study population consisted of women who were enrolled in an ancillary study of the Women's Health Initiative Observational Study (WHI-OS) at WHI clinical centers at Baylor College of Medicine and Wake Forest School of Medicine between February 1995 and July 1998. Women were eligible for the study if they were 50–79 years old, postmenopausal (defined as having had a hysterectomy or not having had menstrual bleeding for the previous 6 months [if ≥56 years old] or 12 months [if 50–55 years old]), planned to live in the area near clinical centers for at least 3 years after study enrollment, and were able to provide written consent. For the purpose of the study, women who self-reported their ethnicity/race as non-Hispanic white and African American (AA) were included. Details of the study rationale and design have been described elsewhere.[29] Of the 834 participants, 9 who did not complete a required baseline screening questionnaire and 213 who had missing information on either the outcome of interest (i.e., ≥3% weight gain since baseline, n = 7) or exposure variables (n = 206) were excluded, resulting in a final study population of 612 women (73% of 834 participants). This study was approved by the institutional review boards at the University of Texas MD Anderson Cancer Center, Baylor College of Medicine, and Wake Forest School of Medicine.

### Data collection

Standardized written protocols and periodic quality assurance visits by the coordinating center were used to assure uniform data collection procedures. At a baseline screening visit, participants completed self-administered questionnaires including demographic and socioeconomic factors, medical and reproductive history, lifestyle behaviors, and general health characteristics. Trained staff performed anthropometric measures including height, weight, and waist and hip circumferences. We assumed that the baseline measurement of the exposure variables had not changed throughout the third annual visit (AV3) unless their measurements were reassessed at AV3 for their changes during the preceding 3 years. In the latter cases (i.e., patients with follow-up measurements at AV3), such as those who smoked or used exogenous estrogen, the AV3 measurements were postulated to dominate the 3-year period; thus, a new variable was created to account for changes in the measurements between baseline and AV3.

Of 42 variables initially selected based on a literature review for their association with obesity and weight gain, after a multicollinearity test (n of variables excluded = 4) and a univariate analysis (n of variables excluded = 11), 27 variables were finally selected for this study. Demographic and socioeconomic characteristics included age, race, and employment status (i.e., full-time, part-time, or unemployed). To evaluate weight gain outcomes, age was classified using 65 as cutoff point of old/young group because in the preliminary CART analysis using weight changes as continuous outcomes, women younger than 65 years had different patterns of weight changes compared with those 65 years or older (average percent weight change = 2.2% in age ≥65 years vs. -0.5% in age < 65 years).

Reproductive history variables included oral contraceptive (OC) use, age at menopause, number of pregnancies, and postmenopausal hormone therapy. Exogenous estrogen use (both opposed and unopposed estrogen use) was classified as never, former, and current; former users were defined as those who stopped estrogen use upon enrollment, and current users included those who began to use estrogen either before or after enrollment and still took the medications at AV3.

Lifestyle variables included smoking status, dietary intake, sleep disturbance, depression, presence or absence of a lifetime sex partner, and physical activity. Data about dietary intake were obtained using the Food Frequency Questionnaire, and only the following variables known to be related to obesity were included: total calories, dietary alcohol and fiber, and percent of calories from saturated fatty acids (SFA), monounsaturated fatty acids (MFA), and polyunsaturated fatty acids (PFA). Metabolic equivalent task (MET) values were assigned for strenuous-, moderate-, and low-intensity activities as 7, 4, and 3 METs, respectively.[15] A total physical activity variable (MET·hours·week^-1^) was then calculated by multiplying the MET level for the activity by the hours exercised per week and summing the values for all types of activities.[30–32] Total physical activity was stratified using 10 METs as a cutoff-point on the basis of current recommendations from the American College of Sports Medicine and the American Heart Association.[31]

General health characteristics measured at AV3 included weight change for the past 2 years when participants were followed up at AV3. The weight change variable was created by subtracting the lowest weight from the highest weight for the previous 2 years. Additionally, lifetime variables of general health included BMI at 35 years and an intentional loss of 10 pounds or more within the past 20 years (except during times when participants were pregnant or sick).

### Outcome variable

The outcome was a binary variable, weight gain. Weight change was estimated by subtracting the weight at baseline from the weight at AV3. The percentage of weight change was calculated by dividing the weight change by the baseline weight (ranged, -24% to 38%) and then classified as less than 3% or 3% or more. The cutoff point of 3% was determined based on the strong consensus about the percentage of weight gain at which risk for obesity-related health effects (e.g., cardiovascular disease and type 2 diabetes) begins to change.[33] Given that weight gain as much as 3% is considered potentially clinically relevant as well as accounting for small weight fluctuations[33], weight gain in our study was defined as 3% or greater of baseline body weight.

### Statistical analysis

Multicollinearity was tested by using coefficient of multiple determination (R2), tolerance, and variance-inflation factor for each exposure variable using remaining covariates as its predictors. Differences in characteristics of participants by weight gain were evaluated using unpaired 2-sample t-tests for continuous variables and chi-square tests for categorical variables. If continuous variables were skewed or had outliers, the Wilcoxon rank-sum test was implemented.

CART analysis was implemented to explore the successive binary divergences of the exposure variables in order to identify subgroups on the basis of their homogeneity in relation to gaining 3% or more weight. The CART built a tree via recursive partitioning and the tree development included three steps: growing the tree, pruning the tree, and validating the tree structure. [12,27,28,34,35] First, a large and complex tree was grown with data from all study variables, each of which was evaluated based on the improvement score using the Gini index in the nominal outcomes (< 3% vs. ≥3% weight gain in our study) and sum of squares in the continuous outcomes (weight changes in our study) to determine the optimum cutoff value (continuous variables) or groupings (nominal variables) that gives the best discrimination between two outcome classes; finally, the strongest predictor variable and its splitting value were determined to split the data into two subgroups (i.e., daughter nodes). The subgroups were then split repeatedly into smaller subgroups representing the most homogeneous split (i.e., terminal node) or daughter nodes in a previous layer. Each terminal node was set to require a minimum of 5 individuals. Because the original tree was too large and statistically uninformative, we then pruned the tree to eliminate branches of the original tree to produce the "right-sized tree," representing the lowest misclassification. Finally, based on the lowest cross-validated error rate, as determined by a cost-complexity pruning algorithm using 10-fold cross-validation, the optimal tree was selected from our pruned trees, which was the best fit and did not over-fit the data. The CART is a nonparametric procedure that does not need any assumptions about the data distribution.[36] Analyses were performed by applying rpart version 4.1–8 for the open-source R statistical software..

Further, stepwise logistic regression was performed to compare findings with those produced by the CART; it produced odds ratios (ORs) and 95% confidence intervals (CIs) of exposure variables for weight gain, stratified by race or age. A 2-tailed *P* value of <0.05 was considered significant. R version 2.15.1 was used.

## Results

Baseline characteristics between < 3% vs. ≥3% weight gain stratified by age (< 65 years vs. ≥65 years) are presented (Table 1). Among women < 65 years, those with ≥3% weight gain were more likely to have undergone an early menopausal transition (*P* = 0.03) and more likely to have intentionally lost 10 pounds or more during the past 20 years (*P* = 0.02). In women 65 years or older, women who gained ≥3% weight were more likely to consume fewer total calories (*P* = 0.02), less dietary alcohol (*P* = 0.04), and less dietary fiber (*P* = 0.003). In both age groups, women with ≥3% weight gain were more likely to have had a greater change in weight during the 2 years prior to AV3 (*P* = 0.001 in the younger group; *P* = 0.02 in the older group). Participants were also stratified by race (AA vs. white women), and their characteristics between < 3% vs. ≥3% weight gain were compared (S1 Table).

**Table 1:** Characteristics of participants enrolled in an ancillary study of the Women’s Health Initiative Observational Study at Baylor College of Medicine or Wake Forest School of Medicine between February 1995 and July 1998.

**Variable**	**Age < 65 years (n = 403)**				**Age ≥65 years (n = 209)**			
	**< 3% weight gain(n = 234)**		**≥3% weight gain(n = 169)**		**< 3% weight gain(n = 154)**		**≥3% weight gain(n = 55)**	
	**n**	**(%)**	**n**	**(%)**	**n**	**(%)**	**n**	**(%)**
**Race**								
***African American***	37	(15.8)	26	(15.4)	22	(14.3)	9	(16.4)
***White***	197	(84.2)	143	(84.6)	132	(85.7)	46	(83.6)
**Occupation**								
***No***	106	(45.3)	75	(44.4)	136	(88.3)	47	(85.5)
***Yes***	128	(54.7)	94	(55.6)	18	(11.7)	8	(14.5)
**Cancer**								
***No***	212	(90.6)	155	(91.7)	126	(81.8)	47	(85.5)
***Yes***	22	(9.4)	14	(8.3)	28	(18.2)	8	(14.5)
**Diabetes**								
***No***	225	(96.2)	161	(95.3)	143	(92.9)	52	(94.5)
***Yes***	9	(3.8)	8	(4.7)	11	(7.1)	3	(5.5)
**Hypertension**								
***No***	160	(68.4)	112	(66.3)	94	(61.0)	34	(61.8)
***Yes***	74	(31.6)	57	(33.7)	60	(39.0)	21	(38.2)
**Cardiovascular disease**								
***No***	203	(86.8)	146	(86.4)	124	(80.5)	39	(70.9)
***Yes***	31	(13.2)	23	(13.6)	30	(19.5)	16	(29.1)
**Oral contraceptive use**								
***No***	136	(58.1)	88	(52.1)	132	(85.7)	46	(83.6)
***Yes***	98	(41.9)	81	(47.9)	22	(14.3)	9	(16.4)
**Exogenous estrogen use**								
***Never***	42	(17.9)	32	(18.9)	57	(37.0)	20	(36.4)
***Former***	18	(7.7)	18	(10.7)	29	(18.8)	7	(12.7)
***Current***	174	(74.4)	119	(70.4)	68	(44.2)	28	(50.9)
**Age at menopause in years, median (range)**	48	(30–64)	46	(30–63)*	50	(32–71)	49	(32–72)
**Number of Pregnancy history**								
***None***	20	(8.5)	9	(5.3)	10	(6.5)	5	(9.1)
***1–2***	93	(39.7)	65	(38.5)	57	(37.0)	14	(25.5)
***≥3***	121	(51.7)	95	(56.2)	87	(56.5)	36	(65.5)
**METs at baseline (METs·hour·week^-1^)**								
***< 10***	172	(73.5)	130	(76.9)	116	(75.3)	40	(72.7)
***≥10***	62	(26.5)	39	(23.1)	38	(24.7)	15	(27.3)
**Sleep disturbance, median (range)** **	13.0	(4.0–18.0)	14.0	(4.0–18.0)	13.0	(5.0–18.0)	14.0	(4.0–17.0)
**Smoking status**								
***Never***	132	(56.4)	84	(49.7)	84	(54.5)	34	(61.8)
***Former***	88	(37.6)	69	(40.8)	65	(42.2)	17	(30.9)
***Current***	14	(6.0)	16	(9.5)	5	(3.2)	4	(7.3)
**Depression** ^†^								
***< 0*.*06***	193	(82.5)	135	(79.9)	140	(90.9)	46	(83.6)
***≥0*.*06***	41	(17.5)	34	(20.1)	14	(9.1)	9	(16.4)
**Lifetime partner**								
***None***	1	(0.4)	2	(1.2)	4	(2.6)	2	(3.6)
***With men or women***	233	(99.6)	167	(98.8)	150	(97.4)	53	(96.4)
**BMI at baseline, kg/m^2^, median (range)**	26.9	(17.9–59.5)	26.9	(16.8–45.5)	25.6	(15.4–51.6)	27.2	(18.3–42.3)
**BMI at 35 years, kg/m^2^, median (range)**	21.9	(13.5–40.7)	22.0	(15.9–59.8)	21.4	(14.0–38.6)	21.8	(17.4–39.5)
**Waist/hip at baseline, ratio, median (range)**	0.789	(0.640–1.095)	0.795	(0.660–1.062)	0.798	(0.663–1.116)	0.796	(0.623–0.984)
**Weight change for the past 2 years, kg, median (range)** ^¶^	4.5	(0.0–37.2)	5.9	(0.0–36.3)*	3.6	(0.0–45.8)	5.0	(0.9–17.2)*
**≥10 pounds lost intentionally within the past 20 years** ^‡^								
***No***	92	(39.3)	47	(27.8)*	76	(49.4)	29	(52.7)
***Yes***	142	(60.7)	122	(72.2)	78	(50.6)	26	(47.3)
**Total calories, kcal, median (range)**	1556	(635–4729)	1482	(642–4087)	1456	(658–4800)	1363	(614–3315)*
**Dietary alcohol, g, median (range)**	0.0	(0.0–83.6)	0.0	(0.0–70.4)	0.0	(0.0–165.9)	0.0	(0.0–23.3)*
**Percent calories from SFA, median (range)**	11.2	(1.9–19.3)	11.1	(5.1–21.3)	10.5	(3.4–18.2)	11.6	(4.6–18.3)
**Percent calories from MFA, median (range)**	12.6	(1.9–23.4)	12.8	(4.3–23.1)	12.0	(5.1–19.9)	13.6	(5.6–18.5)
**Percent calories from PFA, median (range)**	7.2	(2.0–23.4)	7.0	(2.9–17.8)	6.7	(2.1–14.6)	7.6	(2.7–13.2)
**Dietary fiber, g, median (range)**	15.8	(4.8–40.6)	15.8	(4.6–32.9)	16.2	(5.2–38.2)	13.2	(4.6–34.5)*

BMI, body mass index; MET, metabolic equivalent; MFA, monounsaturated fatty acids; PFA, polyunsaturated fatty acids; SFA, saturated fatty acids.

* P < 0.05, chi-square test or Wilcoxon rank-sum test.

** Sleep disturbance score was computed by summing 5 components of relevant questionnaires in the Women’s Health Initiative Observational Study, where a higher score indicates greater sleep disturbance.

^†^ Depression scores were estimated via Burnam's algorithm and categorized using 0.06 as a cutoff-point to detect depressive disorders [43].

^¶^ Weight change was assessed at the third annual visit.

^‡^ Intentional weight loss of more than 10 pounds was examined within the past 20 years, when participants were not pregnant or sick.

### Classification tree

#### Risk profiles of women who gained ≥3% weight, stratified by age

**Fig 1:**
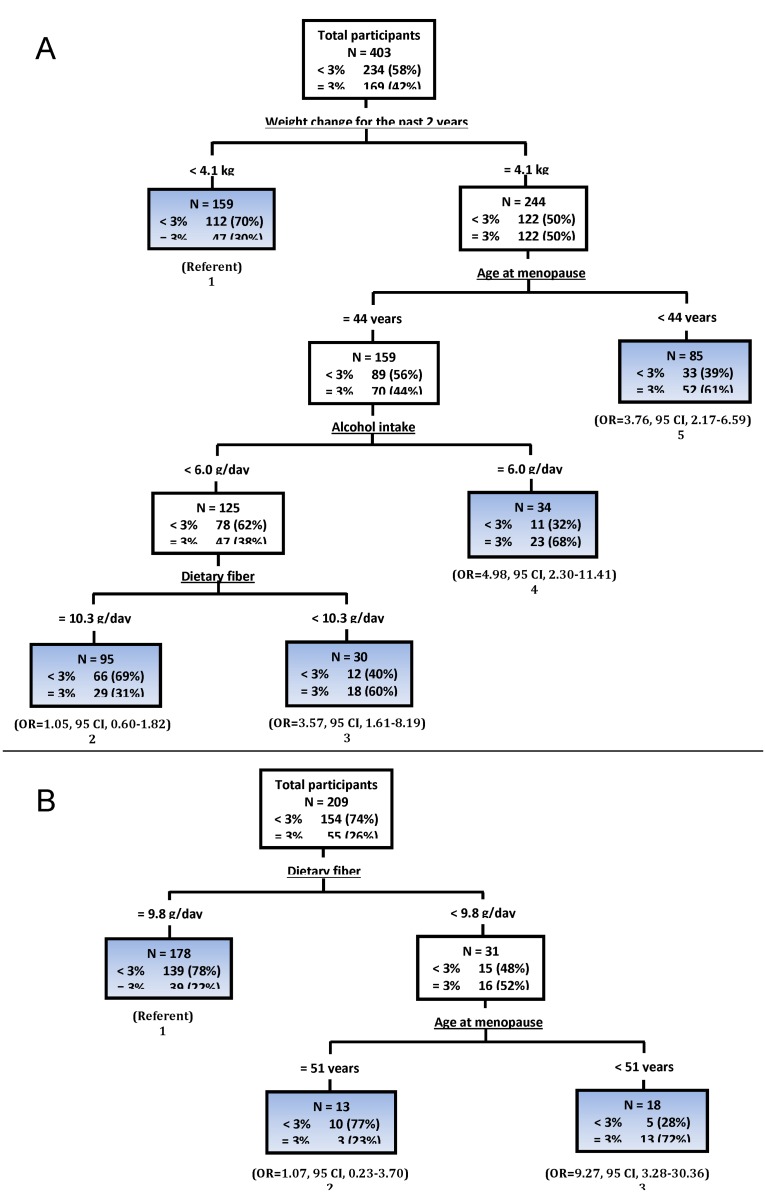
Classification trees identifying profiles of participants who gained ≥3% weight between baseline and the third annual visit in participants, stratified by age (< 65 versus ≥65 years). (CI, confidence interval; OR, odds ratio) ***A***: Participants < 65 years. ***B***: Participants ≥65 years.

In the preliminary classification analysis using weight change as a continuous outcome, age, classified as < 65 years vs. ≥65 years, was identified as the most determinant variable for weight-gain outcome. In addition, the older and younger women differed in the cluster of characteristics associated with ≥3% weight gain. In women < 65 years, the prevalence for gaining ≥3% weight was 42% (Fig 1A). The first split from total participants < 65 years (root node, n = 403) indicating a dominant effect was according to weight change for the past 2 years. With those with < 4.1 kg weight change for the past 2 years as the reference, Fig 1A presents naïve ORs for other terminal nodes. The percentage of women gaining ≥3% weight decreased from 42% (root node) to 30% for women who had < 4.1 kg weight change for 2 years (terminal node 1). Women with ≥4.1 kg weight change during the past 2 years were further split by age at menopause. Compared with women < 4.1 kg weight change for the past 2 years, women who had ≥4.1 kg weight change and entered menopause at < 44 years were more likely to gain ≥3% weight (61%, OR = 3.76, 95% CI, 2.17–6.59, terminal node 5). When women who entered menopause at ≥44 years were further split by alcohol intake and dietary fiber, the percentage of women who gained ≥3% weight increased to 60% for women who consumed < 6 g/day alcohol and < 10.3 g/day dietary fiber (terminal node 3). Women with similar characteristics but who consumed ≥10.3 g/day dietary fiber had decreased risk of gaining ≥3% weight (31%, terminal node 2).

Different patterns are shown in older women (Fig 1B). In women ≥65 years, the percentage of women gaining ≥3% weight was 26%. The first split representing a dominant effect was ≥9.8 g/day dietary fiber intake. With those with ≥9.8 g/day dietary fiber intake as the reference, Fig 1A also shows naïve ORs for other terminal nodes. The lowest risk of gaining ≥3% weight was observed among women who consumed ≥9.8 g/day dietary fiber (22%, terminal node 1). When women who consumed < 9.8 g/day dietary fiber were then split by age at menopause, the prevalence of gaining ≥3% weight increased to 72% for women who entered menopause at < 51 years (OR = 9.27, 95% CI, 3.28–30.36, terminal node 3). On the contrary, women who entered menopause at ≥51 years reduced risk (23%) of gaining ≥3% weight (terminal node 2).

#### Risk profiles of women who gained ≥3% weight, stratified by race

**Fig 2:**
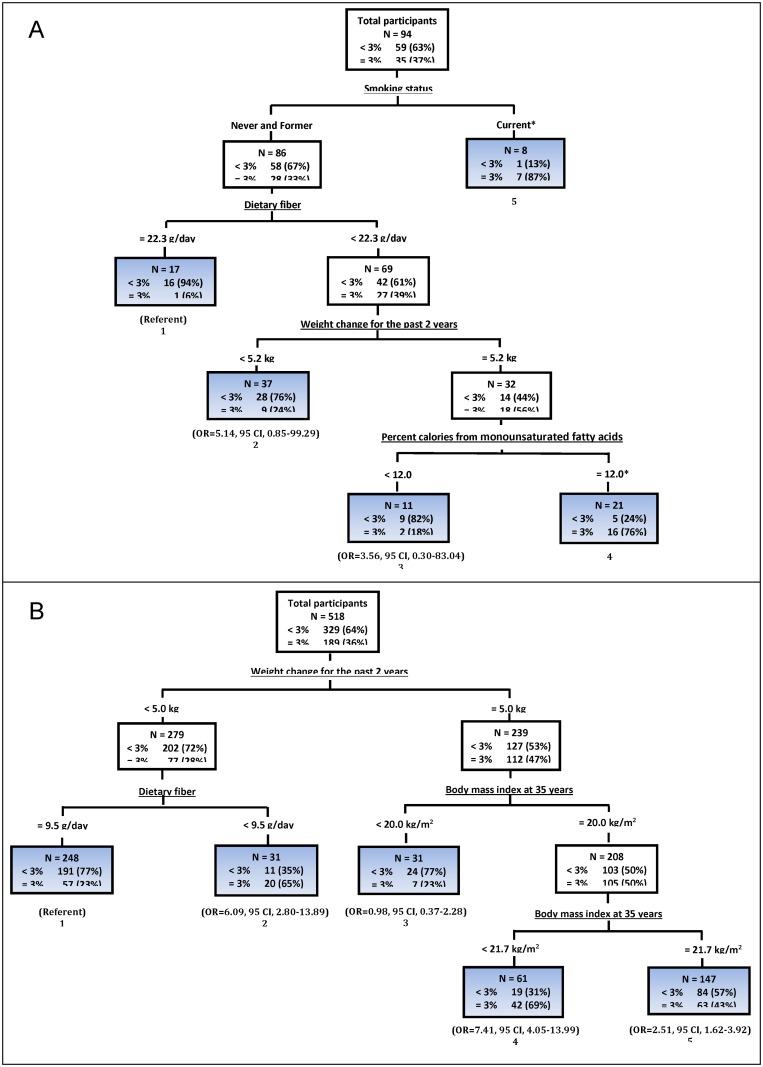
Classification trees identifying profiles of participants who gained ≥3% weight between baseline and the third annual visit in participants, stratified by race (African American women versus white women). (* indicates that due to small sample size, odds ratio and 95% confidence interval could not be converted; CI, confidence interval; OR, odds ratio) ***A***: African American women. ***B***: White women.

Additionally, we identified within racial groups, homogeneous subgroups based on risk characteristics related to ≥3% weight gain. Compared to white women, AA women presented different risk profiles. In AA women, the prevalence of ≥3% weight gain was 37% (Fig 2A), and the dominant variable that split the root node was smoking status. The highest risk of gaining ≥3% weight was observed in women who were current smokers (87%, terminal node 5). Variables that were involved in the next splits were dietary fiber intake, weight change for the past 2 years, and the percentage of calories from MFA. Among never and former smokers, the combination of lower dietary fiber intake, greater weight change for the past 2 years, and higher percentage of calories from MFA increased the risk of ≥3% weight gain (76%, terminal node 4).

Likewise, in white women, the percentage of gaining ≥3% weight was 36% (Fig 2B). The variable demonstrating the greatest impact on ≥3% weight gain was weight change for the past 2 years. Women who had < 5 kg weight change for the past 2 years, when further split, presented different risk patterns according to dietary fiber intake. For example, among women with < 9.5 g/day dietary fiber intake (terminal node 2), the percentage of gaining ≥3% weight increased to 65%; however, women with ≥9.5 g/day dietary fiber intake had decreased risk (23%, terminal node 1). Additionally, women with ≥5 kg weight change for the past 2 years were then split by BMI at 35 years. While women who had < 20 kg/m^2^ BMI at 35 years decreased the risk of ≥3% weight gain to 23% (terminal node 3), women who had ≥20 kg/m^2^ BMI at 35 years increased the risk, but not in a linear pattern; that is, in women who had 20 kg/m^2^ to 21.7 kg/m^2^ BMI at 35 years, the risk of ≥3% weight gain (69%, terminal node 4) were higher than the risk in women with ≥21.7 kg/m^2^ BMI at 35 years (43%, terminal node 5).

### Stepwise regression

The findings from regression analyses were overall comparable to those in CART analyses (Tables 2 and 3). In women < 65 years, weight change for the past 2 years, age at menopause, and dietary fiber intake were related to gaining ≥3% weight; these findings parallel those in the corresponding tree. However, the alcohol intake that emerged in the tree was not significant in the regression analysis. For women ≥65 years, dietary fiber intake, which was the primary splitter in the tree, was found to be the only significant factor in the analysis. Additionally, among AA women, smoking status, dietary fiber intake, and weight change for the past 2 years, involved as splitters in the tree, were significant factors in the stepwise analysis. For white women, all variables that were splitters in the tree (i.e., dietary fiber intake, weight change for the past 2 years, and BMI at 35 years) were also significant in the regression analysis; however, age and BMI at baseline and ≥10 pounds lost intentionally within the past 20 years, which were significant in the regression, were not significant predictors in the CART analysis.

**Table 2:** Stepwise logistic regression odds ratios of 3% or more weight gain, stratified by age (< 65 years vs. ≥65 years) in participants enrolled in an ancillary study of the Women’s Health Initiative Observational Study at Baylor College of Medicine and Wake Forest School of Medicine between February 1995 and July 1998.

**Variable**	**Odds ratio**	**95% CI**	**P-value**
**Age < 65 years**			
** Age at menopause**	0.97	0.94–0.99	0.02
** Dietary fiber**	0.97	0.94–1.00	0.06
** BMI at baseline**	0.92	0.88–0.96	0.001
** BMI at 35 years**	1.06	1.00–1.14	0.06
** Weight change for the past 2 years** *	1.08	1.02–1.14	0.01
** ≥10 pounds lost intentionally within the past 20 years** **			
No	1.00	Referent	
Yes	1.92	1.17–3.18	0.01
**Age ≥65 years**			
** Dietary fiber**	0.93	0.88–0.98	0.01

CI, confidence interval; BMI, body mass index.

* Weight change was assessed at the third annual visit.

** Intentional weight loss of more than 10 pounds was examined within the past 20 years, when participants were not pregnant or sick.

**Table 3:** Stepwise logistic regression odds ratios of 3% or more weight gain, stratified by race (African Americans vs. whites) in participants enrolled in an ancillary study of the Women’s Health Initiative Observational Study at Baylor College of Medicine and Wake Forest School of Medicine between February 1995 and July 1998.

**Variable**	**Odds ratio** *	**95% CI**	**P-value**
**African American women**			
** Diabetes**			
No	1.00	Referent	
Yes	0.20	0.03–0.96	0.06
**Smoking status**			
Never	1.00	Referent	
Former	1.89	0.65–5.66	0.25
Current	31.11	2.91–867.66	0.01
**Dietary fiber**	0.92	0.85–1.00	0.05
**Weight change for the past 2 years** ^†^	1.18	1.03–1.38	0.02
**White women**			
**Age at baseline**			
< 65 years	1.00	Referent	
≥65 years	0.60	0.39–0.93	0.02
**Dietary fiber**	0.97	0.94–1.00	0.06
**BMI at baseline**	0.93	0.89–0.98	0.002
**BMI at 35 years**	1.06	1.00–1.13	0.06
**Weight change for the past 2 years** ^†^	1.05	1.00–1.10	0.03
**≥10 pounds lost intentionally within the past 20 years** **			
No	1.00	Referent	
Yes	1.64	1.04–2.58	0.03

CI, confidence interval; BMI, body mass index.

* In the analysis for African American women, due to scanty sample size, a lifetime partner variable was excluded.

^†^ Weight-change was assessed at the third annual visit.

** Intentional weight loss of more than 10 pounds was examined within the past 20 years, when participants were not pregnant or sick.

## Discussion

Using the CART approach, we sought to construct risk profiles for ≥3% weight gain over 3 years in postmenopausal women within the context of a wide array of modifiable and non-modifiable factors. The individual factors selected in our study have been well documented for their association of obesity and weight gain; however, to our knowledge, such associations in a population of postmenopausal women by clustering these variables into unique risk profiles have not been reported. We demonstrated that when women were stratified by age or race, complex combinations of risk factors differ among subgroups. In addition, the factors that emerged in CART analyses were confirmed using the traditional stepwise regression analyses and the main predictor of weight gain was identified as weight change in the past 2 years for women < 65 years and white women, dietary fiber for women ≥65 years, and smoking status for AA women.

The most dominant factor that predicted weight gain within 3 years in every age and race subgroup was weight change in the past 2 years. Recent studies showed that in postmenopausal women, after weight-loss intervention, weight regain was associated with weight gain after menopause[3,37], indicating that positive weight change (i.e., weight gain) after menopause was a risk factor of regaining weight. Our novel finding was that 5 kg weight fluctuations (including weight gain as well as weight loss) during the past 2 years were related to gaining ≥3% weight at AV3.

In agreement with previous studies[2,3], age at menopause, regardless of the age subgroups, was the next factor increasing the risk of weight gain. Early menopause is associated with weight gain because withdrawal of estrogen reduces lean body mass while increasing fat mass. In addition, compared to premenopausal women, postmenopausal women have a greater ratio of upper body fat to lower body fat.[2] Among postmenopausal women, exogenous estrogen users have been reported to decrease this shift, reducing the risk of obesity-relevant diseases.[2,38] However, we did not observe the significant role of exogenous estrogen use in decreasing the risk of weight gain. Additionally, our subgroup analysis within estrogen users according to duration of hormone use did not show any apparent differences. This is consistent with another study[38] suggesting that hormonal therapy status did not predict postmenopausal weight gain or fat accumulation, rather diminishes the shift of fat from hip to waist.

Across subgroups, the most frequently involved factor in the risk of gaining weight was dietary fiber intake. All participants had a decreased risk of ≥3% weight gain when they consumed 10 g/day or more dietary fiber, except AA women, who had reduced risk if they had ≥22 g/day dietary fiber. Dietary fiber promotes satiety and may reduce energy absorption or stimulate energy expenditure.[19–22]

Smoking has a weight suppressant effect and weight gain is a following result of smoking cessation.[39] For our small subgroup (n = 8) in AA women, on the contrary, the risk of gaining ≥3% weight was 87% in current smokers, which might be an artifact; however, several studies[26,40,41] reporting the positive association between smoking and obesity suggest that the effect of smoking on weight loss is minimal in the short term and rather contribute to obesity and weight gain in the long term.

This study had limitations. The self-reporting of the dietary intake, smoking, and physical activity data limits study conclusions regarding these variables due to the likely prevalence of underreporting of dietary intake and smoking and overreporting of physical activity, especially in obese people. Further studies are warranted to collect data on additional variables, including obesity-relevant genes and biomarkers to increase the accuracy of predictions based on the classification. This study is exclusively based on postmenopausal women, which limits the generalizability to other populations. Additionally, the CART method is exploratory (i.e., it is not based on the probabilistic method), indicating that a composite of trees derived from other populations can be useful to illustrate the possible variability of interactions among risk factors related to weight gain. Despite its shortcomings, CART analysis has advantages. It is well-suited to summarize multiple covariate inter-relationships and provides a simple and easily viewed tree, which is useful for decision making. CART can deal with large numbers of variables and decrease type II errors.[42]

In conclusion, this study revealed that among 27 selected modifiable and non-modifiable variables, greater weight change during the past 2 years, larger than ≥20 kg/m2 body size at 35 years, early menopause, lower intake of dietary fiber, higher intake of fat and alcohol, and smoking were the most relevant factors for gaining ≥3% weight. We used an analytic tree as a means of identifying higher and lower risk groups. Identifying factors related to weight gain within the subgroups of postmenopausal women may allow researchers to target efforts to promote strategies to reduce the risk of obesity and weight gain and maximize the effect of weight control by decreasing obesity-relevant adverse health outcomes.

## Supporting Information

S1 Table Characteristics of participants, stratified by percent weight change according to race, enrolled in an ancillary study of the Women’s Health Initiative Observational Study at Baylor College of Medicine or Wake Forest University School of Medicine between February 1995 and July 1998. (DOCX)
