# Supplementary material for: Risk Profiles for Weight Gain among Postmenopausal Women: A Classification and Regression Tree Analysis Approach
Source: PLoS One. 2015 Mar 30;10(3):e0121430. doi: 10.1371/journal.pone.0121430 (PMC4378852; doi:10.1371/journal.pone.0121430)
Supplement: S1 Table — (DOCX) [file pone.0121430.s001.docx]

Table S1. Characteristics of participants, stratified by percent weight change according to race, enrolled in an ancillary study of the Women’s Health Initiative Observational Study at Baylor College of Medicine or Wake Forest University School of Medicine between February 1995 and July 1998

| **Variable** | **African American women (n= 94)** | | | | | | | | |  | **White women (n= 518)** | | | | | | | | |
| --- | --- | --- | --- | --- | --- | --- | --- | --- | --- | --- | --- | --- | --- | --- | --- | --- | --- | --- | --- |
|  | **< 3% weight gain**  **(n= 59)** | | | |  | **≥ 3% weight gain**  **(n= 35)** | | | |  | **< 3% weight gain**  **(n=329)** | | | |  | **≥ 3% weight gain**  **(n=189)** | | | |
|  | **n** | **(%)** |  |  |  | **n** | **(%)** |  |  |  | **n** | **(%)** |  |  |  | **n** | **(%)** |  |  |
| **Age in years, median (range)** | 60 | (51 | - | 79) |  | 61 | (51 | - | 74) |  | 62 | (50 | - | 79) |  | 60 | (50 | - | 78)* |
| **Occupation** |  |  |  |  |  |  |  |  |  |  |  |  |  |  |  |  |  |  |  |
| **No** | 32 | (54.2) |  |  |  | 20 | (57.1) |  |  |  | 210 | (63.8) |  |  |  | 102 | (54.0)* |  |  |
| **Yes** | 27 | (45.8) |  |  |  | 15 | (42.9) |  |  |  | 119 | (36.2) |  |  |  | 87 | (46.0) |  |  |
| **Cancer** |  |  |  |  |  |  |  |  |  |  |  |  |  |  |  |  |  |  |  |
| **No** | 53 | (89.8) |  |  |  | 28 | (80.0) |  |  |  | 285 | (86.6) |  |  |  | 174 | (92.1) |  |  |
| **Yes** | 6 | (10.2) |  |  |  | 7 | (20.0) |  |  |  | 44 | (13.4) |  |  |  | 15 | (7.9) |  |  |
| **Diabetes** |  |  |  |  |  |  |  |  |  |  |  |  |  |  |  |  |  |  |  |
| **No** | 49 | (83.1) |  |  |  | 32 | (91.4) |  |  |  | 319 | (97.0) |  |  |  | 181 | (95.8) |  |  |
| **Yes** | 10 | (16.9) |  |  |  | 3 | (8.6) |  |  |  | 10 | (3.0) |  |  |  | 8 | (4.2) |  |  |
| **Hypertension** |  |  |  |  |  |  |  |  |  |  |  |  |  |  |  |  |  |  |  |
| **No** | 29 | (49.2) |  |  |  | 14 | (40.0) |  |  |  | 225 | (68.4) |  |  |  | 132 | (69.8) |  |  |
| **Yes** | 30 | (50.8) |  |  |  | 21 | (60.0) |  |  |  | 104 | (31.6) |  |  |  | 57 | (30.2) |  |  |
| **Cardiovascular disease** |  |  |  |  |  |  |  |  |  |  |  |  |  |  |  |  |  |  |  |
| **No** | 49 | (83.1) |  |  |  | 29 | (82.9) |  |  |  | 278 | (84.5) |  |  |  | 156 | (82.5) |  |  |
| **Yes** | 10 | (16.9) |  |  |  | 6 | (17.1) |  |  |  | 51 | (15.5) |  |  |  | 33 | (17.5) |  |  |
| **Oral contraceptive use** |  |  |  |  |  |  |  |  |  |  |  |  |  |  |  |  |  |  |  |
| **No** | 40 | (67.8) |  |  |  | 23 | (65.7) |  |  |  | 228 | (69.3) |  |  |  | 111 | (58.7)* |  |  |
| **Yes** | 19 | (32.2) |  |  |  | 12 | (34.3) |  |  |  | 101 | (30.7) |  |  |  | 78 | (41.3) |  |  |
| **Exogenous estrogen use** |  |  |  |  |  |  |  |  |  |  |  |  |  |  |  |  |  |  |  |
| **Never** | 30 | (50.8) |  |  |  | 11 | (31.4) |  |  |  | 69 | (21.0) |  |  |  | 41 | (21.7) |  |  |
| **Former** | 7 | (11.9) |  |  |  | 5 | (14.3) |  |  |  | 40 | (12.2) |  |  |  | 20 | (10.6) |  |  |
| **Current** | 22 | (37.3) |  |  |  | 19 | (54.3) |  |  |  | 220 | (66.9) |  |  |  | 128 | (67.7) |  |  |
| **Age at menopause in years, median (range)** | 46 | (31 | - | 60) |  | 45 | (30 | - | 61) |  | 50 | (30 | - | 71) |  | 48 | (30 | - | 72)* |
| **Number of pregnancies** |  |  |  |  |  |  |  |  |  |  |  |  |  |  |  |  |  |  |  |
| **None** | 4 | (6.8) |  |  |  | 3 | (8.6) |  |  |  | 26 | (7.9) |  |  |  | 11 | (5.8) |  |  |
| **1-2** | 14 | (23.7) |  |  |  | 15 | (42.9) |  |  |  | 136 | (41.3) |  |  |  | 64 | (33.9) |  |  |
| **≥ 3** | 41 | (69.5) |  |  |  | 17 | (48.6) |  |  |  | 167 | (50.8) |  |  |  | 114 | (60.3) |  |  |
| **METs at baseline (METs·hour·week^-1^)** |  |  |  |  |  |  |  |  |  |  |  |  |  |  |  |  |  |  |  |
| **< 10** | 47 | (79.7) |  |  |  | 33 | (94.3) |  |  |  | 241 | (73.3) |  |  |  | 137 | (72.5) |  |  |
| **≥ 10** | 12 | (20.3) |  |  |  | 2 | (5.7) |  |  |  | 88 | (26.7) |  |  |  | 52 | (27.5) |  |  |
| **Sleep disturbance, median (range)**** | 14.0 | (4.0 | - | 18.0) |  | 14.0 | (5.0 | - | 18.0) |  | 13.0 | (4.0 | - | 18.0) |  | 14.0 | (4.0 | - | 18.0) |
| **Smoking status** |  |  |  |  |  |  |  |  |  |  |  |  |  |  |  |  |  |  |  |
| **Never** | 37 | (62.7) |  |  |  | 16 | (45.7)* |  |  |  | 179 | (54.4) |  |  |  | 102 | (54.0) |  |  |
| **Former** | 21 | (35.6) |  |  |  | 12 | (34.3) |  |  |  | 132 | (40.1) |  |  |  | 74 | (39.2) |  |  |
| **Current** | 1 | (1.7) |  |  |  | 7 | (20.0) |  |  |  | 18 | (5.5) |  |  |  | 13 | (6.9) |  |  |
| **Depression†** |  |  |  |  |  |  |  |  |  |  |  |  |  |  |  |  |  |  |  |
| **< 0.06** | 48 | (81.4) |  |  |  | 27 | (77.1) |  |  |  | 285 | (86.6) |  |  |  | 154 | (81.5) |  |  |
| **≥ 0.06** | 11 | (18.6) |  |  |  | 8 | (22.9) |  |  |  | 44 | (13.4) |  |  |  | 35 | (18.5) |  |  |
| **Lifetime partner** |  |  |  |  |  |  |  |  |  |  |  |  |  |  |  |  |  |  |  |
| **None** | 0 | (0) |  |  |  | 1 | (2.9) |  |  |  | 5 | (1.5) |  |  |  | 3 | (1.6) |  |  |
| **With either women or with men** | 59 | (100) |  |  |  | 34 | (97.1) |  |  |  | 324 | (98.5) |  |  |  | 186 | (98.4) |  |  |

Table S1 (Continued)

| **Variable** | **African American women (n= 94)** | | | | | | | | |  | **White women (n= 518)** | | | | | | | | |
| --- | --- | --- | --- | --- | --- | --- | --- | --- | --- | --- | --- | --- | --- | --- | --- | --- | --- | --- | --- |
|  | **< 3% weight gain**  **(n= 59)** | | | |  | **≥ 3% weight gain**  **(n= 35)** | | | |  | **< 3% weight gain**  **(n=329)** | | | |  | **≥ 3% weight gain**  **(n=189)** | | | |
|  | **n** | **(%)** |  |  |  | **n** | **(%)** |  |  |  | **n** | **(%)** |  |  |  | **n** | **(%)** |  |  |
| **BMI at baseline, kg/m^2^, median (range)** | 29.7 | (20.7 | - | 51.6) |  | 28.9 | (18.4 | - | 45.5) |  | 25.6 | (15.4 | - | 59.5) |  | 26.7 | (16.8 | - | 45.3) |
| **Waist/hip at baseline, ratio, median (range)** | 0.807 | (0.696 | - | 0.964) |  | 0.819 | (0.732 | - | 0.948) |  | 0.790 | (0.640 | - | 1.116) |  | 0.793 | (0.623 | - | 1.062) |
| **Weight change for the past 2 years, kg, median (range)¶** | 4.1 | (0.9 | - | 18.1) |  | 5.4 | (0.0 | - | 19.1) |  | 4.1 | (0.0 | - | 45.8) |  | 5.4 | (0.0 | - | 36.3)* |
| **BMI at 35 years, kg/m^2^, median (range)** | 22.9 | (13.5 | - | 40.7) |  | 22.0 | (16.7 | - | 33.3) |  | 21.6 | (14.0 | - | 39.1) |  | 21.8 | (15.9 | - | 59.8) |
| **≥ 10 pounds lost intentionally within the past 20 years‡** |  |  |  |  |  |  |  |  |  |  |  |  |  |  |  |  |  |  |  |
| **No** | 21 | (35.6) |  |  |  | 13 | (37.1) |  |  |  | 147 | (44.7) |  |  |  | 63 | (33.3)* |  |  |
| **Yes** | 38 | (64.4) |  |  |  | 22 | (62.9) |  |  |  | 182 | (55.3) |  |  |  | 126 | (66.7) |  |  |
| **Total calories, kcal, median (range)** | 1513 | (670 | - | 4800) |  | 1242 | (614 | - | 3734) |  | 1528 | (635 | - | 4729) |  | 1422 | (652 | - | 4087) |
| **Dietary alcohol, g, median (range)** | 0.0 | (0.0 | - | 16.5) |  | 0.0 | (0.0 | - | 17.4) |  | 0.0 | (0.0 | - | 165.9) |  | 0.0 | (0.0 | - | 70.4) |
| **Percent calories from SFA, median (range)** | 11.2 | (1.9 | - | 18.5) |  | 11.6 | (6.7 | - | 15.7) |  | 10.7 | (3.4 | - | 19.3) |  | 11.2 | (4.6 | - | 21.3) |
| **Percent calories from MFA, median (range)** | 12.4 | (1.9 | - | 22.2) |  | 14.1 | (9.1 | - | 20.1)* |  | 12.4 | (4.2 | - | 23.4) |  | 12.7 | (4.3 | - | 23.1) |
| **Percent calories from PFA, median (range)** | 7.4 | (2.2 | - | 13.8) |  | 8.1 | (4.0 | - | 15.9) |  | 7.0 | (2.0 | - | 23.4) |  | 7.0 | (2.7 | - | 17.8) |
| **Dietary fiber, g, median (range)** | 16.3 | (5.2 | - | 34.0) |  | 11.9 | (4.6 | - | 32.9)* |  | 15.9 | (4.8 | - | 40.6) |  | 15.5 | (4.6 | - | 34.5) |

BMI, body mass index; MET, metabolic equivalent; MFA, monounsaturated fatty acids; PFA, polyunsaturated fatty acids; SFA, saturated fatty acids

* P < 0.05, chi-square test or Wilcoxon rank-sum test.

** Sleep disturbance score was computed by summing 5 components of relevant questionnaires in the Women’s Health Initiative Observational Study, where a higher score indicates greater sleep disturbance.

† Depression scores were estimated via Burnam's algorithm and categorized using 0.06 as a cutoff-point to detect depressive disorders.^43^

¶ Weight change was assessed at the third annual visit.

‡ Intentional weight loss of more than 10 pounds was examined within the past 20 years when participants were not pregnant or sick.
